# Abnormal neurofilament inclusions and segregations in dorsal root ganglia of a Charcot-Marie-Tooth type 2E mouse model

**DOI:** 10.1371/journal.pone.0180038

**Published:** 2017-06-27

**Authors:** Jian Zhao, Kristy Brown, Ronald K. H. Liem

**Affiliations:** 1 Department of Pathology and Cell Biology, Columbia University College of Physicians & Surgeons, New York, New York, United States of America; 2 Department of Pathology and Cell Biology and Taub Institute for Research in Alzheimer’s Disease and the Aging Brain, Columbia University College of Physicians & Surgeons, New York, New York, United States of America; University of Missouri Columbia, UNITED STATES

## Abstract

Charcot-Marie-Tooth (CMT) disease or hereditary motor and sensory neuropathy is the most prevalent inherited peripheral neuropathy and is associated with over 90 causative genes. Mutations in neurofilament light polypeptide gene, *NEFL* cause CMT2E, an axonal form of CMT that results in abnormal structures and/or functions of peripheral axons in spinal cord motor neurons and dorsal root ganglion neurons. We have previously generated and characterized a knock-in mouse model of CMT2E with the N98S mutation in *Nefl* that presented with multiple inclusions in spinal cord neurons. In this report, we conduct immunofluorescence studies of cultured dorsal root ganglia (DRG) from *Nefl*^*N98S/+*^ mice, and show that inclusions found in DRG neurites can occur in embryonic stages. Ultrastructural analyses reveal that the inclusions are disordered neurofilaments packed in high density, segregated from other organelles. Immunochemical studies show decreased NFL protein levels in DRG, cerebellum and spinal cord in *Nefl*^*N98S/+*^ mice, and total NFL protein pool is shifted toward the triton-insoluble fraction. Our findings reveal the nature of the inclusions in *Nefl*^*N98S/+*^ mice, provide useful information to understand mechanisms of CMT2E disease, and identify DRG from *Nefl*^*N98S/+*^ mice as a useful cell line model for therapeutic discoveries.

## Introduction

Charcot-Marie- Tooth (CMT) diseases are the most common inherited sensory and motor neuropathies with a reported prevalence of 1 in 2,500 people worldwide [1]. Nerves outside the brain and spinal cord are damaged, causing muscle weakness and numbness in the legs and arms. There have been over 90 genes identified to cause CMT. CMT is divided into two major types, CMT1 and CMT2, based on nerve conduction velocities. CMT1 is primarily a demyelinating neuropathy and has a reduced conduction velocity, whereas CMT2 is largely an axonal neuropathy and has a fairly normal conduction velocity.

Neurofilament light polypeptide gene (*NEFL*) has been identified as the causative gene in CMT2E [2]. The *NEFL* gene encodes neurofilament light polypeptide (NFL), one of the neuronal intermediate filaments proteins (IFs) that form the major structural framework that maintains the diameter of the axon and thus the normal transmission of nerve signals. Neuronal IFs consist of the neurofilament triplet proteins (NFL, NFM and NFH for neurofilament light, middle and high), α-internexin and peripherin [3]. Like all intermediate filaments, each monomer of NFL protein is composed of an α-helical rod domain flanked by N-terminal head domain and C-terminal tail domain [3]. The α-helical rod domain is necessary for the formation of a coiled-coil dimer, which is the first step in intermediate filament assembly. Human NFL can self-assemble in the absence of other intermediate filament proteins, but forms a much more elaborate network when either NFM or NFH is present [4]. In vivo, NFL is always present along with NFM throughout development, whereas NFH appears somewhat later [5]. Currently, over 18 mutations in the *NEFL* gene have been linked to CMT disease (Human intermediate filament database: http://www.interfil.org/details.php?id=NM_006158). Although these mutations are generally considered to cause CMT2E, there are cases of patients with an *NEFL* mutation who present with a slower nerve conduction velocity resembling CMT1F [6, 7]. Two autosomal recessive *NEFL* mutations have also been reported, causing truncated NFL proteins resulting in a severe form of CMT [8, 9].

The N98S mutation is located at the beginning of the rod domain, and transfection experiments have shown that the normal intermediate filament network is disrupted both in the absence and presence of NFM [10]. The N98S mutation was first described in sporadic cases, and was characterized by early-onset sensorimotor neuropathy with variable clinical features including motor delay, mental retardation, hearing loss, nystagmus and cerebellar ataxia [8, 11–13]. Two recent familial cases have been reported with slowing NCV close to the demyelinating or intermediate CMT, and delayed motor milestones with complex clinical features including cerebellar ataxia [11].

We have established a knock-in *Nefl*^*N98S/+*^ mouse model that recapitulates most of the features of patients with this mutation [14]. The animals had a tremor and showed decrease in balance. Immunohistochemical analyses showed multiple inclusions in the cell bodies and axons of spinal cord neurons and dorsal root ganglia, disorganized processes in the cerebellum and abnormal processes in the cerebral cortex and pons [2]. However, details of the nature of these inclusions are lacking. Here we have conducted electron microscopy and immunocytochemical studies on cultured dorsal root ganglia from adult mice in order to get a better understanding of the composition of these inclusions and try to unravel the mechanism of the disease.

## Results

### Neurofilament accumulations in cultured dorsal root ganglia neurons from adult *Nefl*^*N98S/+*^ mice

**Fig 1:**
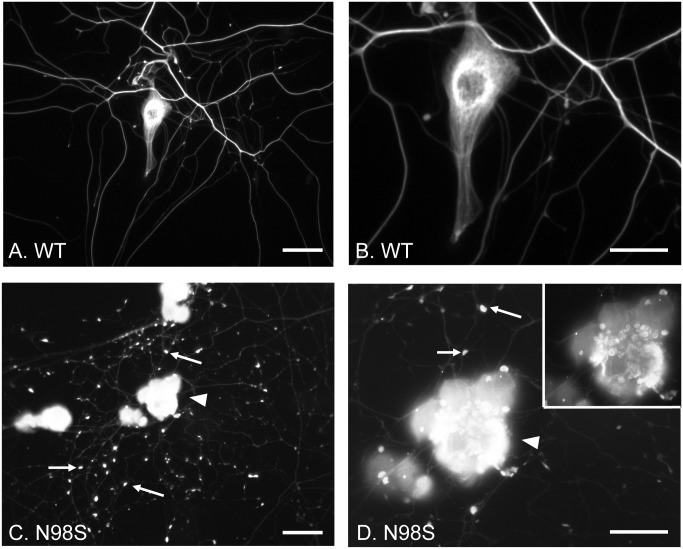
Immunofluorescence micrographs of cultured DRG neurons from *Nefl*^*+/+*^ and *Nefl*^*N98S/+*^ mice labeling NFL. (A and C) Low power views of DRG neurons from *Nefl*^*+/+*^ (A) and *Nefl*^*N98S/+*^ (C) mice. Note that in *Nefl*^*N98S/+*^ DRG neurons, the processes are characterized by large amounts of enlarged and bright particles along the processes, pointed by arrows (C). (B and D) High-magnification images of DRG neurons as seen in Fig A and C. Note the filamentous structure in *Nefl*^*+/+*^ DRG neuron (B). In contrast, disrupted neurofilament network and enlarged neurofilament particles (pointed by arrows) along the processes can be seen in *Nefl*^*N98S/+*^ DRG neurons (D). A low intensity image of DRG neurons pointed by the arrowhead is shown in the inset (D) that also shows the broken neurofilamentous network. *Nefl*^*N98S/+*^, n = 8; *Nefl*^*+/+*^, n = 5. Scale bars = 50 μm (A and C) and 25 μm (B and D).

We have previously observed the presence of neurofilamentous inclusions in the cell bodies and processes of the dorsal root ganglia (DRG) of the *Nefl*^*N98S/+*^ mice (14). We performed immunocytochemical studies of cultured DRG neurons from 4-month-old mice with anti-NFL antibody (Fig 1). Cultured DRG neurons from *Nefl*^*+/+*^ mice showed cell body staining and continuous process labeling (Fig 1A). At a higher power, filamentous structures were observed in the cell body (Fig 1B). In contrast, DRG neurons from *Nefl*^*N98S/+*^ mice showed strong cell body labeling and a punctate pattern of process labeling, where a number of enlarged and abnormally intense punctae were labeled (pointed by arrows). The inset is a low exposure image of the cell body pointed by the arrowhead. At higher magnification, neurofilament accumulations could be seen in the cell body and the filamentous structure seen in *Nefl*^*+/+*^ was disrupted in *Nefl*^*N98S/+*^ DRG neurons (Fig 1D). In addition, the staining of the processes from the cultured *Nefl*^*N98S/+*^ DRGs was very weak relative to the cell body staining when compared to the processes and cell bodies of the cultured *Nefl*^*+/+*^ DRGs (Fig 1).

### Abnormal neurofilament accumulations in cultured embryonic DRG explants from *Nefl*^*N98S/+*^ mice

**Fig 2:**
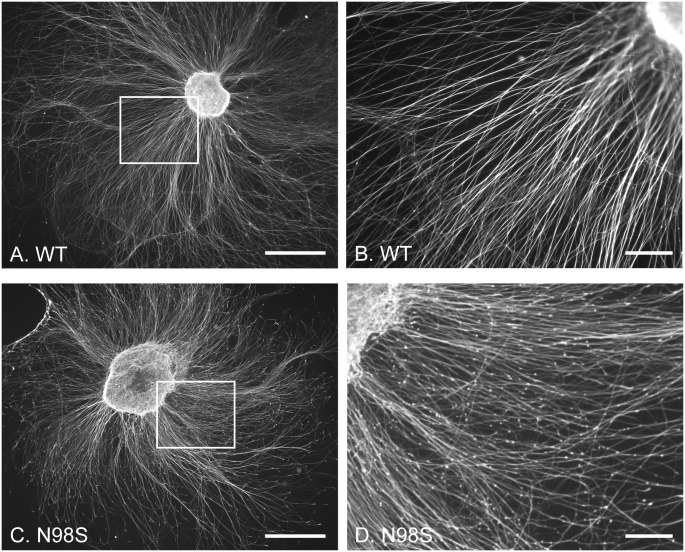
Immunofluorescence micrographs of cultured DRG explants from wild-type and *Nefl*^*N98S/+*^ mice embryos (E15) labeling NFL. (A and C) Low power views of DRG explants from *Nefl*^*+/+*^ (A) and *Nefl*^*N98S/+*^ (C) embryos cultured on the laminin-coated coverslip for 3 days. (B) Higher magnification of the boxed area of wild-type DRG in Fig A. Note the processes are labeled quite uniformly with anti-NFL antibody. (D) Higher magnification of the boxed area of *Nefl*^*N98S/+*^ DRG in Fig C. Note the processes are characterized by a large amount of bright particles along the processes. 3 *Nefl*^*+/+*^ females bred with *Nefl*^*N98S/+*^ males were sacrificed, and embryos were collected and genotyped. DRG explants from those embryos were cultured. Embryos for *Nefl*^*+/+*^, n = 10. Embryos for *Nefl*^*N98S/+*^, n = 11. Scale bars = 500 μm (A and C) and 100 μm (B and D).

We also compared the expression pattern of neurofilaments in embryonic DRG explants from *Nefl*^*+/+*^ and *Nefl*^*N98S/+*^ mice embryos at E15 *in vitro* (Fig 2). By using embryonic DRG explants, the original architecture of the DRG is maintained with preserved interactions between DRG neurons with non-neuronal cells; additionally, it provides useful information of *Nefl*^*N98S/+*^ embryos during development. After 72 h in culture, *Nefl*^*+/+*^ DRG explants extended tightly fasciculated bundles of axons, which showed a pattern of continuous labeling with anti-NFL antibody (Fig 2A and 2B). *Nefl*^*N98S/+*^ DRG explants extended a network of axons that did not show any obvious morphological abnormalities at low magnification (Fig 2A and 2C). However, at higher magnification a punctate pattern was observed for *Nefl*^*N98S/+*^ axons, where a lot of brighter and enlarged punctae were observed (Fig 2D). This punctate pattern of *Nefl*^*N98S/+*^ embryonic DRG explants is reminiscent of that in dissociated DRG neurons from *Nefl*^*N98S/+*^ adult mice (Fig 1C and 1D), indicating that abnormal neurofilament organization in *Nefl*^*N98S/+*^ DRGs may occur as early as E15 embryos.

### Electron microscopy of DRG from adult mice

**Fig 3:**
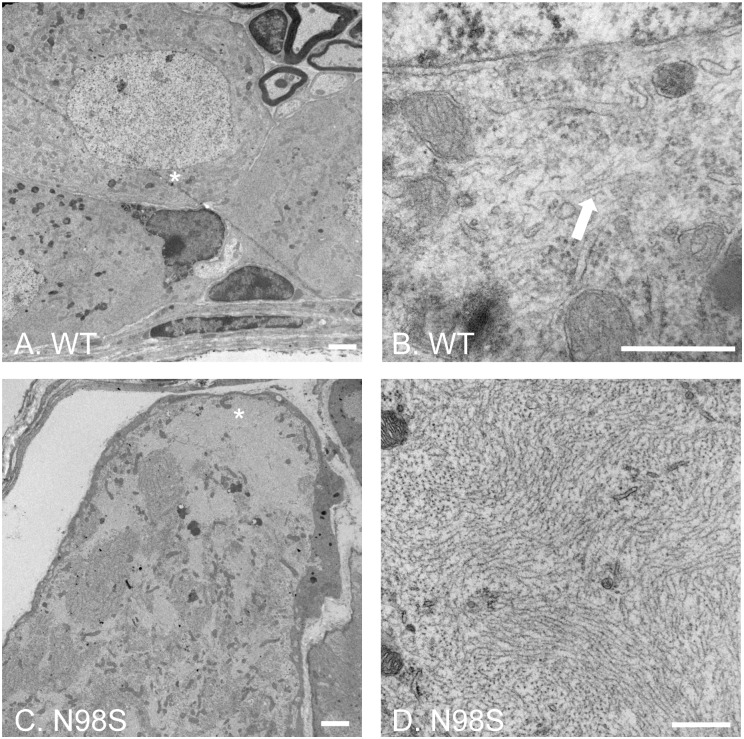
Electron micrographs of DRG cell soma of wild-type and *Nefl*^*N98S/+*^ mice. (A and C) Low power views of cell soma of DRG from wild-type (A) and *Nefl*^*N98S/+*^ (C) mice. Note the large amount of aggregates throughout the soma of *Nefl*^*N98S/+*^ DRG (C). (B and D) High power views of asterisk-labeled areas in Fig A and C. The filamentous structure formed by intermediate filaments is pointed by the arrow in *Nefl*^*+/+*^ DRG (B). Massive accumulation of disordered neurofilaments is observed in *Nefl*^*N98S/+*^ DRG (D). The density of neurofilaments is very high and few other cytoplasmic elements can be seen within the aggregates. Scale bars = 2 μm (A and C) and 500 nm (B and D).

The NFL-positive inclusions were observed in cultured DRGs in vitro are similar to the immunohistochemical staining of DRGs from adult *Nefl*^*N98S/+*^ mice (14). To determine the nature of these neurofilamentous inclusions, we performed an electron microscopic study of DRGs from adult mice. In the low-power images of DRG cell soma (Fig 3A and 3C), there was an apparent difference in the ultrastructural organization of the cell body region, where numerous electron-lucent patches were observed throughout the soma of *Nefl*^*N98S/+*^ DRG (Fig 3C). Higher-power image of the asterisk-labeled region of Fig 3A showed that in *Nefl*^*+/+*^ DRGs, filamentous structures formed by individual intermediate filaments can be seen in the soma (pointed by arrow) (Fig 3B). In contrast, a high-power image of the asterisk-labeled electron-lucent region in Fig 3C showed massive accumulations of disordered neurofilaments packed together in high density. Few other cytoplasmic components were seen within the accumulations in *Nefl*^*N98S/+*^ DRG cell soma (Fig 3D). This EM result is consistent with the immunohistochemical result of DRG from our previous studies, showing neurofilament aggregates in *Nefl*^*N98S/+*^ DRG cell body at the light microscopic level [14]. The results show that these aggregates consist of bundles of neurofilaments, rather than amorphous aggregates.

**Fig 4:**
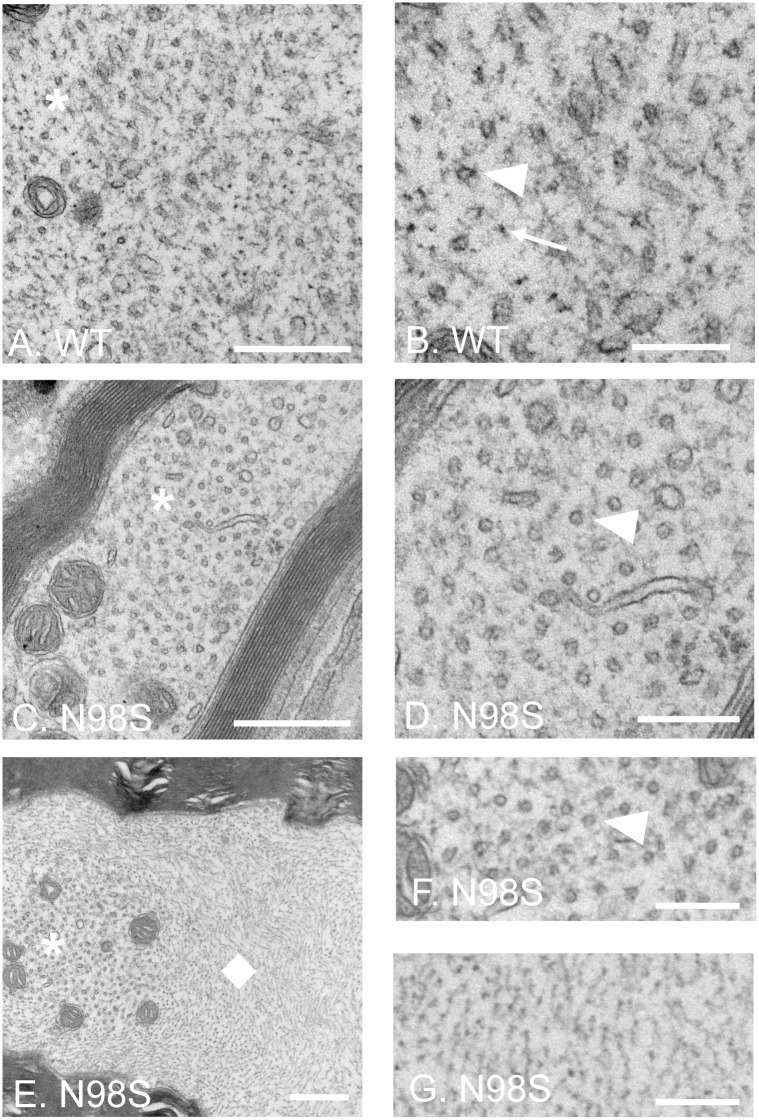
Electron micrographs of cross sections of DRG axons of wild-type and *Nefl*^*N98S/+*^ mice. (A, C and E) Low power views of DRG axons from *Nefl*^*+/+*^ (A) and *Nefl*^*N98S/+*^ (C and E) mice. (B) A high power view of the asterisk-labeled area in *Nefl*^*+/+*^ DRG axons from Fig A. Microtubules (pointed by an arrowhead) and neurofilaments (pointed by an arrow) are interspersed. (D) A high power view of the asterisk-labeled area in one cross section of *Nefl*^*N98S/+*^ DRG axons from Fig C. Neurofilaments are barely seen and replaced by more microtubules (pointed by an arrowhead). (F) A high power view of the asterisk-labeled area in another cross section of *Nefl*^*N98S/+*^ DRG axons from Fig E. Microtubules (pointed by an arrowhead) and barely any neurofilaments can be seen in this area. (G) A high power view of the diamond-labeled area in another cross section of *Nefl*^*N98S/+*^ DRG axons from Fig E. Neurofilaments are packed at high density in this area, almost exclusive of any other organelles. (E, F and G) Microtubules and other organelles are segregated from the neurofilament aggregates in this cross section of *Nefl*^*N98S/+*^ DRG axons. Scale bars = 500 nm (A, C and E) and 200 nm (B, D, F and G).

The axons of the DRGs were also examined by EM (Fig 4). Both microtubules and neurofilaments were in the axons of *Nefl*^*+/+*^ DRGs, and neurofilaments were distributed among microtubules and other organelles (Fig 4A and 4B). Distinct patterns of cytoskeletal organization were observed from axons of *Nefl*^*N98S/+*^ DRG (Fig 4C–4G). In one transverse section of an axon, few neurofilaments were found; instead, microtubules were the predominant structures in the axon of *Nefl*^*N98S/+*^ DRG (Fig 4C and 4D). In another section of an axon under low power, mitochondria and some other organelles were surrounded by some fiber-like structures of high density (Fig 4E). A high power view of the asterisk-labeled area showed that mitochondria and microtubules were found in that area with few neurofilaments (Fig 4F). In contrast, in another area, we observed what appeared to be bundles of intermediate filaments (Fig 4E) surrounded by microtubules and other organelles. A high power view of the area pointed by a diamond confirmed the presence of neurofilaments that formed the massive accumulations (Fig 4G).

### Electron microscopy of cultured DRG neurons from adult mice

**Fig 5:**
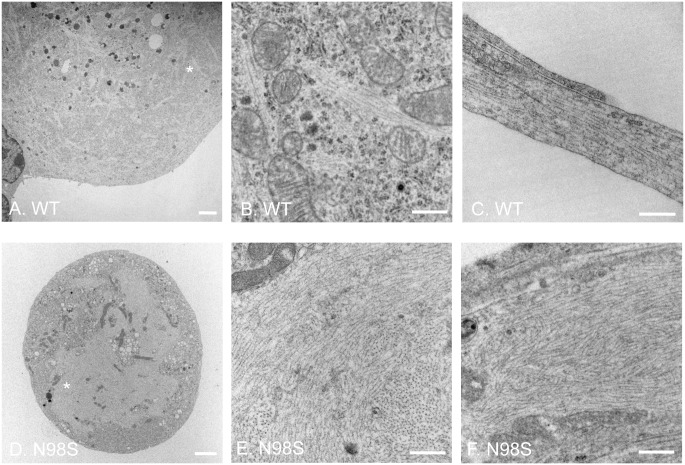
Electron micrographs of cultured DRG neurons from wild-type and *Nefl*^*N98S/+*^ mice. (A and B) EM of cell soma of wild-type. Fig B is a higher magnification view of the asterisk-labeled area in Fig A. Filamentous structures formed by intermediate filaments can been seen throughout the cell body, next to organelles such as mitochondria. (C) A longitudinal section of processes of *Nefl*^*+/+*^ DRG. Bundles of microtubules and intermediate filaments are running in parallel alongside the processes. (D and E) EM of cell soma of *Nefl*^*N98S/+*^ DRG. Fig E is a higher magnification view of the asterisk-labeled area in Fig D. Massive accumulation of disordered neurofilaments is observed in the soma of *Nefl*^*N98S/+*^ DRG (D). The density of neurofilaments is very high and few other cytoplasmic elements can be seen within the accumulations (E). (F) A longitudinal section of processes of *Nefl*^*N98S/+*^ DRG. An enlarged process area of disorganized filamentous accumulation can be seen. Scale bars = 2 μm (A and D) and 500 nm (B, C, E and F).

To get a better understanding of the punctate pattern of the *Nefl*^*N98S/+*^ DRG cultures observed in Fig 1, electron microscopy was performed on cultured DRG neurons (Fig 5). A lower magnification image of *Nefl*^*+/+*^ DRGs showed that electron-lucent bundles form a network-like structure throughout the cell body region (Fig 5A). A higher magnification image of the asterisk-labeled area revealed that the electron-lucent bundles consisted of filamentous structures, which were believed to be neurofilaments (Fig 5B). In *Nefl*^*N98S/+*^ DRG neurons, however, the electron-lucent bundles disappeared. Instead, a large electron-lucent mass accumulated in the center of the cell body, and was segregated from the electron-dense cytosolic components in the *Nefl*^*N98S/+*^ DRG neuron (Fig 5D). A higher power image of the asterisk-labeled area showed that the large electron-lucent mass was composed almost exclusively of disorganized neurofilaments, in the absence of other cellular components (Fig 5E). Longitudinal sections of processes from *Nefl*^*+/+*^ and *Nefl*^*N98S/+*^ DRG neurons were also examined using electron microscopy. Neurofilaments (filamentous structures with a smaller diameter) and microtubules (larger diameter) ran in parallel bundles in the processes of *Nefl*^*+/+*^ DRGs (Fig 5C). However, in one image of the *Nefl*^*N98S/+*^ DRGs, the process was swollen and was filled with large numbers of neurofilaments (Fig 5F). The massive accumulations of neurofilaments found in enlarged processes of *Nefl*^*N98S/+*^ DRGs would almost certainly be strongly stained by NFL antibodies in immunofluorescence. This result is consistent with the finding of many bright and enlarged punctae labeled by anti-NFL antibody in Fig 1C.

### Neurofilament proteins in *Nefl*^*N98S/+*^ mice are reduced in both Triton-X 100 soluble and insoluble fractions

**Fig 6:**
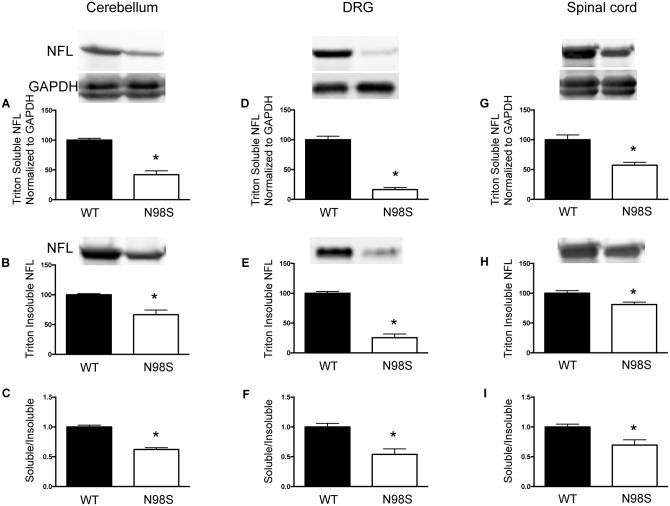
*Nefl*^*N98S/+*^ mice showing reduced protein levels of NFL in both triton-X 100 soluble and triton-X 100 insoluble fractions of cerebellum, DRG and spinal cord. A, D and G) Quantitative data from western blots of triton soluble fraction of cerebellum, DRG and spinal cord, respectively. The intensity of NFL was normalized to that of GAPDH. NFL levels in *Nefl*^*+/+*^ were further normalized to 100. (B, E and H) Quantitative data from western blots of triton insoluble fraction of cerebellum, DRG and spinal cord, respectively. NFL levels in *Nefl*^*+/+*^ were normalized to 100. (C, F and I) The ratio of triton soluble NFL to triton insoluble NFL in cerebellum, DRG and spinal cord, respectively. The ratio of *Nefl*^*+/+*^ was normalized to 100. A litter of 4 *Nefl*^*+/+*^ and 4 *Nefl*^*N98S/+*^ mice was used in this study. * *p* < 0.05.

We have previously examined the total protein levels of NFL from brain, spinal cord and sciatic nerve of *Nefl*^*N98S/+*^ mice, and found a decrease in NFL protein levels in all three tissues [14]. As N98S NFL proteins formed massive accumulations in DRGs, we assessed changes in NFL solubility in the cerebellum, DRG and spinal cord using Triton-X 100 (Fig 6). We were interested in determining whether the percentage of Triton-X100 soluble protein decreased in the mutant animals, which might suggest that the aggregates are less soluble. We found that all three tissues from *Nefl*^*N98S/+*^ mice showed a reduction in Triton-X 100 soluble NFL proteins (Fig 6A, 6D and 6G). Triton soluble NFL proteins in *Nefl*^*N98S/+*^ DRGs had the greatest decrease to 16% (84% decrease) of that of *Nefl*^*+/+*^ (Fig 6D), and exhibited a 58% decrease in cerebellum (Fig 6A) and a 43% decrease in spinal cord (Fig 6G). In the Triton insoluble fractions, *Nefl*^*N98S/+*^ DRGs showed a 75% decrease in NFL protein compared to *Nefl*^*+/+*^ (Fig 6E), whereas cerebellum and spinal cord of *Nefl*^*N98S/+*^ exhibited a 33% and a 20% decrease compared to *Nefl*^*+/+*^, respectively (Fig 6B and 6H).

The ratio of soluble- to insoluble-fraction of NFL proteins was calculated for each tissue by dividing the normalized soluble NFL proteins to the insoluble NFL proteins. The ratio in all the three tissues of *Nefl*^*N98S/+*^ mice is significantly lower than that of *Nefl*^*+/+*^ (Fig 6C, 6F and 6I), suggesting that the insoluble NFL proteins account for a larger portion of the total NFL protein pool in *Nefl*^*N98S/+*^ mice.

## Materials and methods

### *Nefl*^*N98S/+*^ mice

*Nefl*^*N98S/+*^ mice were generated in our laboratory as described previously[14]. The animals are maintained in a C57BL6 background obtained from Jackson Laboratory. The mice have been deposited at Jackson Laboratory and are available for use upon request. Mice were maintained in sterilized micro-isolator cages housed in virus-free barrier facilities, and fed with autoclaved food and water. All animal studies were performed according to a protocol approved by the Institutional Care and Use Committee (IACUC) at Columbia University Medical Center.

### Primary neuronal cultures

For dissociated adult dorsal root ganglia neurons, mice of 3–6 months were euthanized by CO_2_ exposure followed by cervical dislocation. DRGs were collected from the spinal column and transferred to ice-cold HBSS. Roots and nonneuronal tissue were trimmed from DRGs with fine forceps and a micro knife. Trimmed DRGs were incubated with 1.25 mg/ml collagenase for 90 min at 37°C, 5% CO_2_, and incubated with 0.25% trypsin for 5 min at 37°C. DRGs were washed and resuspended in complete medium (see below), and were dissociated into single cells by gentle trituration. A cushion of 2 ml 15% BSA was slowly added to the bottom of the cell suspension to remove debris through a centrifugation step of 200 x g for 10 min. For immunofluorescence, DRG neurons were plated on coverslips pre-coated with poly-D-lysine (100 μg/ml, Sigma, P-0899) and laminin (5 μg /ml, Sigma, L-2020) for 2 h at 37°C and 5% CO_2_ to allow adherence. Then wells were flooded with complete medium consisting of Ham’s F-12 medium, 10% horse serum, 2 mM L-glutamine, 100 U/ml penicillin, 100 μg /ml streptomycin, 50 μM 5-fluoro-2’-deoxyuridine and 150 μM uridine. For electron microscopy, DRG neurons were plated on 8-well Permanox chamber slides (Nunc).

For embryonic DRG explants, DRGs were dissected from individual embryonic day 15 (E15) *Nefl*^*+/+*^ and *Nefl*^*N98S/+*^ littermates. DRG explants were placed on coverslips pre-coated with poly-D-lysine and laminin, and grown in neurobasal medium supplemented with B27 (Invitrogen), 2 mM L-glutamine, 1 mM Na pyruvate and 100 ng/ml NGF. DRG explants were grown for 72 h at 37°C and 5% CO_2_ before they were fixed for immunofluorescence studies.

### Immunofluorescence

Adult DRG neurons or embryonic DRG explants grown on coverslips for 72 h were fixed with 4% paraformaldehyde (PFA) in PBS (pH 7.4) for 15 min at room temperature. The coverslips were washed twice with PBST (0.1% Triton x-100 in PBS) and blocked with 10% goat serum in PBST for 30 min. Overnight incubation was performed at 4°C with an anti-NFL primary antibody described before [15]. The following day, coverslips were washed three times with PBST and incubated for 1 h with AlexaFluor-488-goat anti-rabbit IgG (H+L). Coverslips were washed three times with PBST and mounted using Aquamount. Images were taken with a Zeiss Axioplan 2 microscope equipped with camera AxioCam with Axiovision software (Zeiss).

### Electron microscopy

For DRG explants, adult mice were anesthetized and perfused with 4% PFA and 2% glutaraldehyde in 0.1 M cacodylate buffer (pH 7.4). DRGs were immersed in the same fixative for 24 h at 4°C, washed with cacodylate buffer and post-fixed in 1% osmium tetroxide for 1 h. For dissociated DRG neurons, cells were cultured for 72 h before they were fixed with 2.5% glutaraldehyde in 0.1 M Sorensen’s buffer for 1 h. After three washes with Sorensen’s buffer, DRGs were post fixed in 1% osmium for 1h. They were then dehydrated in a graded series of ethanol and embedded in EMS-812 resin. Plastic sections (1 μm) were stained with toluidine blue and examined for light microscopy. Ultrathin thin sections were cut on the PT-XL ultramicrotome at 60nm thick. The sections were stained with uranyl acetate and lead citrate and examined under a JEOL JEM-1200 EXII electron microscope. Images were captured with an ORCA-HR digital camera (Hamamatsu) and recorded with an AMT Image Capture Engine.

### Western blotting

Cerebellum, DRG or spinal cord tissues were homogenized in ice-cold phosphate-buffered saline (PBS) containing 1% triton X-100, sonicated and centrifuged for 30 min in 15000 rpm at 4°C. The supernatant was transferred to a new tube; the pellet was resuspended with PBS containing 1% triton X-100, and processed again following the above steps. The supernatants were combined together and used as triton X-100 soluble fraction. The pellet was resuspended in a buffer containing 8 M urea, 2% sodium dodecyl sulfate (SDS), 50 mM Tris (pH 8.0), 50 mM Na_2_HPO_4_ and 300 mM NaCl. After resuspension and centrifugation, the supernatant was taken out to use as triton X-100 insoluble fraction.

Protein concentrations of the soluble fraction were determined using the Coomassie Protein Assay kit (Thermo Fisher Scientific, Waltham, MA, USA). 20 μg of cerebellum, DRG and spinal cord proteins were separated by SDS-PAGE and transferred to Immobilon-P membrane (Millipore). The membranes were blocked with 10% fat-free milk in PBST and incubated with primary antibodies in blocking solution overnight at 4°C. The anti-NFL primary antibody was the same as the one used in immunofluorescence. Anti-GAPDH antibody was purchased from EnCor Biotechnology Inc. (Gainesville, FL, USA). After washing with PBST for three times, the membrane was incubated with secondary antibodies for 1 h and washed with PBST for three times. IRDye^®^ 680RD Goat anti-Rabbit IgG (H+L) (LICOR) and 827–08364 IRDye 800CW Goat anti-Mouse IgG (H+L) (LICOR) were used as secondary antibodies. The immunoreactive proteins were visualized with an Odyssey infrared scanner (LICOR Biosciences). Intensities of the blot signals were measured in arbitrary units using ImageJ. Soluble NFL protein levels were normalized to GAPDH. NFL protein levels of *Nefl*^*N98S/+*^ mice were compared to WT NFL protein levels, which were set to 100. Western blot of a dilution series of the sample stained with anti-NFL was performed to confirm the linearity of the range of NFL examined in this assay (S1 Fig). Animals were from the same litter of 4 *Nefl*^*+/+*^ mice and 4 *Nefl*^*N98S/+*^ mice. Statistical significance was set to *p* < 0.05 and calculated using unpaired t-test.

## Discussion

Abnormal neurofilament accumulations are observed in many neurodegenerative diseases. They have also been observed in a number of mouse models with mutations or overexpression of neuronal intermediate filaments [4, 14, 16–22]. These neurofilament accumulations appear to lead to neuronal dysfunction and/or loss of neurons. Transgenic mice overexpressing alpha internexin showed axonal swelling and neurofilament misaccumulations in Purkinje cells, and progressive loss of Purkinje neurons, as well as motor coordination deficits that preceded the loss of the Purkinje neurons [4]. Transgenic mice overexpressing peripherin lacking NFL expression (Per;NFL -/-) have neurofilament inclusions in both perikarya and axons of motor neurons, whereas another mouse model overexpressing hNFH in NFL null background (hNFH;NFL -/-) led to nonfilamentous aggregates only in the perikarya of motor neurons [16, 23]. The difference in the nature of the inclusions in these two mouse models and their different localizations may lead to different consequences: the neurofilamentous inclusions in neurites of Per;NFL -/- mice overexpressing peripherin are correlated with motor neuron death, whereas the hNFH;NFL -/- mice developed motor dysfunction such as tremors and muscle weakness, but did not suffer motor neuron death [16, 23]. In order to model the *NEFL* mutation in CMT2E, we have generated a knock-in mouse with a N98S mutation in *Nefl* [14]. In this study, we demonstrated that neurofilament inclusions in DRGs of *Nefl*^*N98S/+*^ mice could occur as early as embryos at E15. These inclusions were found in both the soma and axons of the DRGs. We have studied in more detail the cytoskeletal organization in the DRGs and the nature of the filaments in these inclusions and found that rather than non-filamentous aggregates, they consist of bundles of filaments. We hypothesize that these inclusions could potentially be toxic and lead to the degeneration of these neurons. We believe that chemical compounds that help remove the inclusions would be able to alleviate disease phenotypes and bring a partial rescue.

One striking difference in cytoskeletal organization in DRG axons between *Nefl*^*+/+*^ and *Nefl*^*N98S/+*^ mice is the segregation of neurofilaments and other organelles (Fig 4E). Similar segregations have been described in sural nerve of CMT2E patients [24], motor neurons of a transgenic mouse expressing a mutant NFL [20], GAN [25], and other neuropathies induced by β, β’-iminodiproprionitrile (IDPN) [26, 27], 2,5-hexanedione [28] and aluminum [29, 30]. However, at least two questions remain unanswered. Firstly, what is the underlying mechanism of neurofilament-microtubule segregation, and second, what is the relationship between the cytoskeletal segregation and the neurofilament accumulations. Brown and colleagues have developed a stochastic model to explain how the segregation occurred [31]. They propose that the neurofilament-microtubule segregation is a consequence of impaired neurofilament transport, and the rate and extent of this segregation is dependent on the extent to which neurofilament transport is inhibited [31]. But it is still not clear how neurofilament accumulations arise. It has been suggested that neurofilament-microtubule segregation precedes the neurofilament accumulation after IDPN administration [26]. Considering the decreased level of N98S NFL proteins, it is likely that the N98S mutation destabilizes the mutant NFL protein and leads to an impaired transport of neurofilament polymer, which causes the segregation of neurofilaments and microtubules, resulting in neurofilament accumulations and axonal swelling. The disorganized cytoskeleton in axons could0 further inhibit cargo transport along axons, eventually leading to CMT2E neuropathy.

The reduced protein levels in both the soluble and insoluble fractions of *Nefl*^*N98S/+*^ mice are consistent with our previous finding of reduced total NFL (wild-type + mutant) protein levels in *Nefl*^*N98S/+*^ mice [14]. It should be noted, however, that the soluble and insoluble fractions derived from our method do not necessarily represent an exhaustive separation of all small subunits and polymers of neurofilaments. Nevertheless, it has been found that polymerized neurofilament proteins forming cytoskeletons are mostly insoluble to conserve the cytoskeletal organization [32]. Therefore, the decreased level of insoluble neurofilaments in *Nefl*^*N98S/+*^ mice indicates fewer polymerized neurofilaments in the cytoskeleton, which might affect cytoskeletal structure and function. The lower soluble/insoluble ratio in *Nefl*^*N98S/+*^ mice suggests that N98S mutation tilts the soluble/insoluble balance towards the insoluble, mostly in the form of accumulations. These data combined with our previous results of decreased total protein levels and unchanged mRNA levels in tissues of *Nefl*^*N98S/+*^ mice suggest that faster degradation of the mutant proteins than wild-type NFL contributes, at least partially, to the decreased NFL protein levels (wild-type plus mutant) in the *Nefl*^*N98S/+*^ mice.

In summary, our results demonstrate that the neurofilament proteins from the *Nefl*^*N98S/+*^ mice form inclusions in the processes and cell bodies of DRG neurons and DRG explants cultured in vitro. These inclusions were already observed in DRG explant cultures from embryonic day 15 mice. Electron microscopy studies reveal that the inclusions are predominantly disordered neurofilaments packed in high density, almost exclusive of anything else. In *Nefl*^*N98S/+*^ mice, both the triton soluble and insoluble portions of NFL proteins are reduced. Among the total NFL pool, the soluble-insoluble balance shifts toward the triton-insoluble fraction.

## Supporting information

S1 Fig A series of diluted samples from wild-type spinal cord were analyzed by western blotting using the rabbit anti-NFL antibody as described in the Materials and Methods section. Following scanning, linear regression was performed on the samples to confirm the linearity of the range of NFL examined in this assay. Protein amounts: sample 1, 30 μg; sample 2, 27 μg; sample 3, 24 μg; sample 4, 21 μg; sample 5, 18 μg; sample 6, 15 μg; sample 7, 12 μg; sample 8, 9 μg; sample 9, 6 μg; sample 10, 3 μg.
(TIF)
