# Supplementary figures and images for: Abnormal neurofilament inclusions and segregations in dorsal root ganglia of a Charcot-Marie-Tooth type 2E mouse model
Source: PLoS One. 2017 Jun 27;12(6):e0180038. doi: 10.1371/journal.pone.0180038 (PMC5487060; doi:10.1371/journal.pone.0180038)

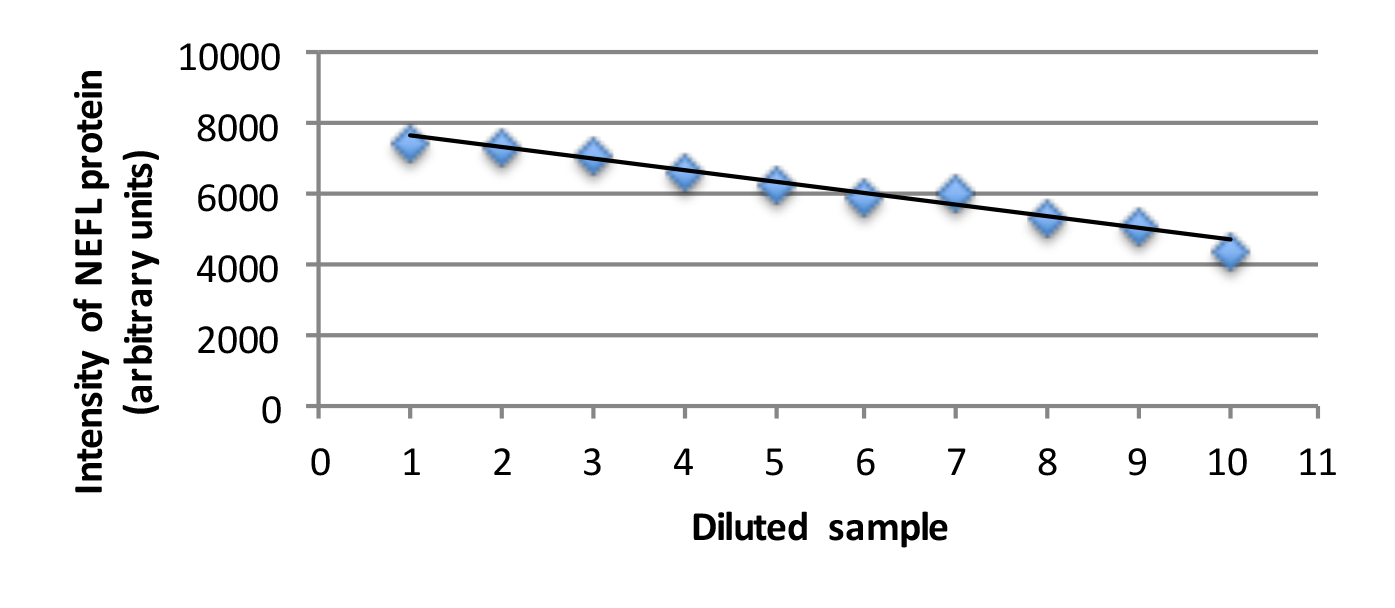

Supplement: S1 Fig — Following scanning, linear regression was performed on the samples to confirm the linearity of the range of NFL examined in this assay. Protein amounts: sample 1, 30 μg; sample 2, 27 μg; sample 3, 24 μg; sample 4, 21 μg; sample 5, 18 μg; sample 6, 15 μg; sample 7, 12 μg; sample 8, 9 μg; sample 9, 6 μg; sample 10, 3 μg. (TIF) [file pone.0180038.s001.tif]
